# Pilot Randomised Trial of a Brief Online Personalised Feedback Intervention for the UK Context Designed To Prevent, Reduce, and Address Gambling Harm

**DOI:** 10.1007/s10899-025-10401-2

**Published:** 2025-06-27

**Authors:** John A. Cunningham, David C. Hodgins, Stephen Sharman, Hollie Walker, Christina Schell

**Affiliations:** 1 National Addiction Centre, Institute of Psychiatry, Psychology and Neuroscience, King’s College London, London, UK; 2 Institute for Mental Health and Policy Research, Centre for Addiction and Mental Health, Toronto, ON Canada; 3 Department of Psychiatry, University of Toronto, Toronto, ON Canada; 4 Department of Psychology, University of Calgary, Calgary, Canada

**Keywords:** Gambling, Personalized normative feedback, Randomized controlled trial, Internet intervention

## Abstract

Participation in online gambling is growing and the risk of experiencing harms is also increasing. Brief personalised feedback interventions have been shown to prevent, reduce and address gambling harm and this randomised controlled trial tested the effectiveness of a version customised for the UK. A sample of 1586 online gambling participants with moderate or problem gambling were rapidly recruited from an existing Internet panel of UK residents. Participants were randomised to a no intervention control group or received the self-directed, online intervention which included normative feedback and personalised information explaining the consequences of gambling above lower-risk guidelines. One- and three-month follow-ups assessed the short-term impact on frequency and harm. Feedback and recommendations were collected to guide improvements and increase future utility. All gambling outcomes showed improvement between the initial survey and both follow-ups, however, there were no differences between the intervention and control groups. Most participants displayed normative misperceptions when estimating how much others the same age and gender gambled. The majority of the sample had never previously sought treatment despite over a third of these reporting moderate or problematic levels of gambling. There is need for a publicly available, low-cost alternative to traditional treatment in order to help the large proportion of people with gambling concerns who would not otherwise seek formal support. Although an intervention effect was not detected in this sample, Internet-based alternatives remain a promising opportunity meriting further research.

**Supplementary Information:**

The online version contains supplementary material available at 10.1007/s10899-025-10401-2.

## ClinicalTrial.gov

NCT06011070.

**Supplementary Information:**

The online version contains supplementary material available at 10.1007/s10899-025-10401-2.

## Introduction

People who engage in at-risk or disordered gambling can cause substantial harms to themselves and society (Blank et al., 2021). The large majority of individuals will never access treatment, despite experiencing concerns (Cunningham, 2005; Slutske, 2006) and can face a number of barriers to seeking treatment. Some examples include limited problem recognition, shame associated with seeking help, the impression that face-to-face treatment is a poor match for their risky gambling, and limited knowledge regarding alternative options for accessing care (Suurvali et al., 2012).

The UK has a relatively permissive gambling environment with a multitude of gambling opportunities available at all times via online and land-based gambling. Accordingly, gambling is a common activity in the UK; recent figures indicate that approximately 48% of adults 18 years of age or older have gambled in the past 4 weeks, and individuals are more likely to engage with gambling online, than in person (Wardel et al., 2024). The most recent figures from the UK Gambling Commission indicate that of those who have gambled in the previous 12 months, 4.1% score in the ‘problem’ gambling category on the Problem Gambling Severity Index (PGSI) (Wardel et al., 2024).

Other work has identified interest among people with risky and disordered gambling for access to self-directed materials to help them address their gambling-related harm without seeking formal treatment (Cunningham et al., 2008). A substantial amount of work has been devoted to designing and evaluating self-directed interventions for people with risky gambling (Hodgins & Makarchuk, 2002) and online versions are an important addition to existing health service options. Online interventions have the advantage of being accessible any time and allow the delivery of help without requiring face-to-face contact, which remains a concern in the post-pandemic era. Given the increased prevalence of online gambling (Gambling Commission, 2021), the development of these interventions is particularly well timed. Furthermore, by reducing barriers these interventions have the potential to promote greater social equality through more accessible care and increasing the wide-spread availability of effective, self-directed online interventions could help prevent, reduce, and address gambling harms.

A recent systematic review showed some early support that online interventions were effective in reducing gambling harms (Sagoe et al., 2021). Of the 13 studies reviewed, four related to work optimising a brief, online intervention for Canadians (Cunningham et al., 2012) that provided personalised feedback to participants by comparing their reported gambling to age and gender matched group averages. The intent of this type of personalised feedback intervention is to motivate change by promoting social comparison with others (Cunningham et al., 2012) and through an assessment of current risk. Previous work with similar interventions, both for gambling (Cunningham et al., 2012) and unhealthy alcohol consumption (Cunningham et al., 2009) have found that these interventions can promote small reductions in the target behaviour. Given that the intervention is designed to be brief, relevant to a wide range of people with gambling concerns, and to be distributed widely and free-of-charge, even small reductions have the potential for significant public health impact.

One potentially important addition to such interventions is the inclusion of lower-risk gambling guidelines. These guidelines, adapted from Canadian research and tailored to the UK context (Hodgins et al., 2022; Rochester & Cunningham, 2022), provide evidence-based benchmarks that quantify the threshold above which gambling-related harm becomes more likely. Their inclusion may help individuals better contextualise their behaviour not only relative to others (normative feedback), but also against empirically derived thresholds for harm. This may be especially useful for those who do not perceive themselves as problem gamblers but are still engaging in high-risk behaviours.

A public, online intervention is not currently available in the UK. To this end, a previously trialled normative feedback intervention (Cunningham et al., 2012) was modified to provide feedback based on UK norms data. New to this version of the intervention, the feedback contained personalised feedback regarding lower-risk gambling recommendations. As the lower-risk gambling recommendations have been generated to be relevant to many countries globally, an intervention with feedback on lower-risk guidelines found effective in the UK setting would also have relevance to other countries. This randomised controlled trial (RCT) investigated the short-term impact of the customised UK intervention in reducing gambling frequency and the experience of related harms among people with moderate risk or problem gambling.

## Methods

The study design was a two-arm, parallel group, randomised controlled trial with 1- and 3-month post-randomisation follow-ups.

### Recruitment

Recruitment occurred through the Prolific website with advertisements targeting members who voluntarily indicated that they participate in online gambling (not including online lotteries) (Peer et al., 2017). While there are potential limitations associated with recruitment for intervention trials using online panels (Cunningham et al., 2019), they have the advantage of allowing for rapid recruitment. Further, compared to general population samples, there is a higher likelihood of panel participants reporting problem gambling, specifically due to higher rates of online gambling (Sturgis & Kuha, 2022). Recruitment occurred in two stages. First, individuals who answered the advertisement were asked to ‘complete a short survey about gambling,’ and that they would be compensated £2 for their time. Links to the information sheet and consent form were then provided. Those who consented were asked to complete the baseline questionnaire, which included questions to determine eligibility for the randomised trial. In the second stage, eligible individuals were asked if they were interested in completing an additional study to help with the development and evaluation of tools for people concerned about their gambling and were told that participation would include two additional surveys which would be sent after 1- and 3-months with a £3 honorarium for each completed survey. Participants were told that they would be contacted to complete the follow-up surveys through the Prolific website. Those who were not eligible or who declined to participate in the additional study were thanked for their time and paid.

### Eligibility Criteria

Individuals who (1) were age 18 or older; (2) correctly answered the attention check questions on the baseline survey; (3) stated they answered all questions truthfully; (4) confirmed they live in the UK; and (5) reported gambling in a fashion indicating moderate risk or problem gambling (i.e. scored three or more on the Problem Gambling Severity Index (PGSI) (Ferris & Wynne, 2001) were eligible for the trial.

### Randomisation

During the consent stage, participants were told that not everyone would receive the same materials. After agreeing to participate in the additional study, participants were randomised in a 1:1 ratio by an automated algorithm programmed in the survey platform (i.e., the research team were not involved in the automated randomization process). Once allocated to either the Intervention or No Intervention condition, participants were immediately provided with the corresponding material.

### Experimental Groups

#### Intervention Condition

A brief assessment of the participant’s gambling (i.e. single item asking frequency of any gambling activity, number of different types of gambling activities reported, PGSI) was used to generate the intervention’s feedback report, which was divided into four sections: (1) normative feedback comparing the participant’s gambling to others from the UK of the same age and gender; (2) personalised feedback regarding the risk of harm based on frequency of gambling, and of number of different types of gambling the participant engaged in; (3) a report of the PGSI score for the participant along with an interpretation of the score; and (4) summary of choices about incorporating lower-risk gambling recommendations in the future. The intervention also encouraged people with significant concerns to take steps towards seeking further treatment. An anonymous copy of the online intervention can be found at (https://qualtrics.kcl.ac.uk/jfe/form/SV_3xG5M7d4kFn928C).

#### No Intervention Condition

Participants in the control group (No Intervention condition) completed the brief assessment but were not provided with any intervention materials. Instead, participants were given a list of the different components of the current online intervention and were asked to think about how useful they would find each component. The rationale for using this control group was to provide a control group that, while not containing an active intervention, would make sense to participants who had been asked to an additional study to help with the development and evaluation of tools for people concerned about their gambling.

To the best of our knowledge, there were no technological issues experienced during the conduct of this study.

### Assessment & Outcome Measures

During the initial assessment, participants supplied demographic information (e.g. age, gender, education, employment, socioeconomic status), completed a series of questions related to their gambling (e.g. gambling activities, treatment history), and completed the PGSI. The PGSI assesses an individual’s risk of experiencing consequences related to gambling activities, with higher scores indicating greater risk. The scores can also be categorised as: non-problem (0), low-risk (1–2), moderate risk (3–7), and problem gambling (8+).

The primary outcome measures collected asked about: (1) mean time spent gambling (hours) and amount of money spent on gambling in a typical month (as participants lost money on average, this variable is referred to as expenditures; from the past 3 months for the baseline and 3-month follow-up survey; in the past month for the 1 month follow-up survey); (2) reported harm from one’s own gambling using the Short Gambling Harm Screen (SGHS) (Browne et al., 2018); and (3) the severity of urges to gamble using the Gambling Symptom Assessment Scale (G-SAS) (Kim et al., 2009). The SGHS assesses commonly reported harms of excessive gambling. This can help identify individuals who do not otherwise meet clinical criteria, but whose gambling is nonetheless impacting their personal well-being (Browne et al., 2018). The SGHS is comprised of 10-items, each measured in a yes/no format (score range 0–10). The G-SAS is a 12-item tool that measures the severity of gambling symptoms across three domains: urges, thoughts, and gambling behaviour. For this study the four questions relating to urges were utilised and scored from 0 (no symptoms) to 4 (extreme symptoms), with total scores ranging from 0 to 16. These measures were collected at baseline and again during the 1- and 3-month follow-up assessments.

Normative perceptions were collected during each survey by asking participants to estimate the frequency of gambling others their same age and gender participated in. Finally, during the 1-month follow-up survey, participants in the intervention group were asked to provide feedback about the intervention. Participants were asked how useful they found the current version, if there were any elements that could be made easier to understand, and what other elements they might find helpful.

### Sample Size Estimate

Using specifications derived from other research involving personalised feedback interventions (Cunningham et al., 2012), the sample size was calculated to test if the modified intervention had a small impact (d = 0.20) on frequency of gambling (i.e. roughly a 2 day per month reduction) with an alpha level of 0.05 and power of 0.80. It was determined that a sample of 787 participants would be needed to detect a small effect (estimated using GPower). An 85% follow-up rate at 3-months was assumed based on rates achieved by a recently completed alcohol use study which recruited from Prolific (91% and 84% follow-up at 1- and 6-months respectively). This meant 926 participants eligible for the randomised trial would need to be recruited at baseline and a conservative estimate was made that 74 participants might decline (*n* = 1,000 participants).

### Data Analysis Plan

#### Gambling Outcomes

To assess the impact of the intervention on gambling harm, linear regression mixed-effects models with random intercepts were conducted for each of the primary and secondary outcome variables. More specifically, each of the six models independently examined the main effects of time, condition assignment, and the time by condition assignment interaction on the primary outcome measures. Missing data was estimated using a restricted maximum likelihood approach. Analyses were repeated including a variable indicating greater gambling severity at baseline as a fixed effect (Problem Gambling Severity Index (PGSI) scores ≥ 7) in order to determine if controlling for differences in participants’ risky gambling at baseline resulted in better fitting models.

#### Normative Perceptions

Linear regression mixed-effects models with random intercepts were also used to analyse participant estimates of the time and amount of money an average person their age and gender spends on gambling during a typical month and session. The resulting four models independently examined the main effects of time and condition assignment and the corresponding interaction (i.e., time by condition assignment) on participant’s normative estimates. A restricted maximum likelihood approach was used to estimate missing data. All analyses were conducted using SPSS version 27.0 (*IBM SPSS Statistics for Windows*, 2022).

#### UK Intervention Feedback

During the 1-month follow-up survey, participants in the Intervention condition were asked five questions about the utility of the feedback elements (i.e., comparison of types of gambling, likelihood of experiencing problems from gambling based on types, likelihood of experiencing problems based on frequency, PGSI score, suggestions for how to reduce risk of harm). Comments and feedback were summarised and features that could benefit from further modification were noted.

## Results

### Recruitment and Follow-Up

**Fig. 1:**
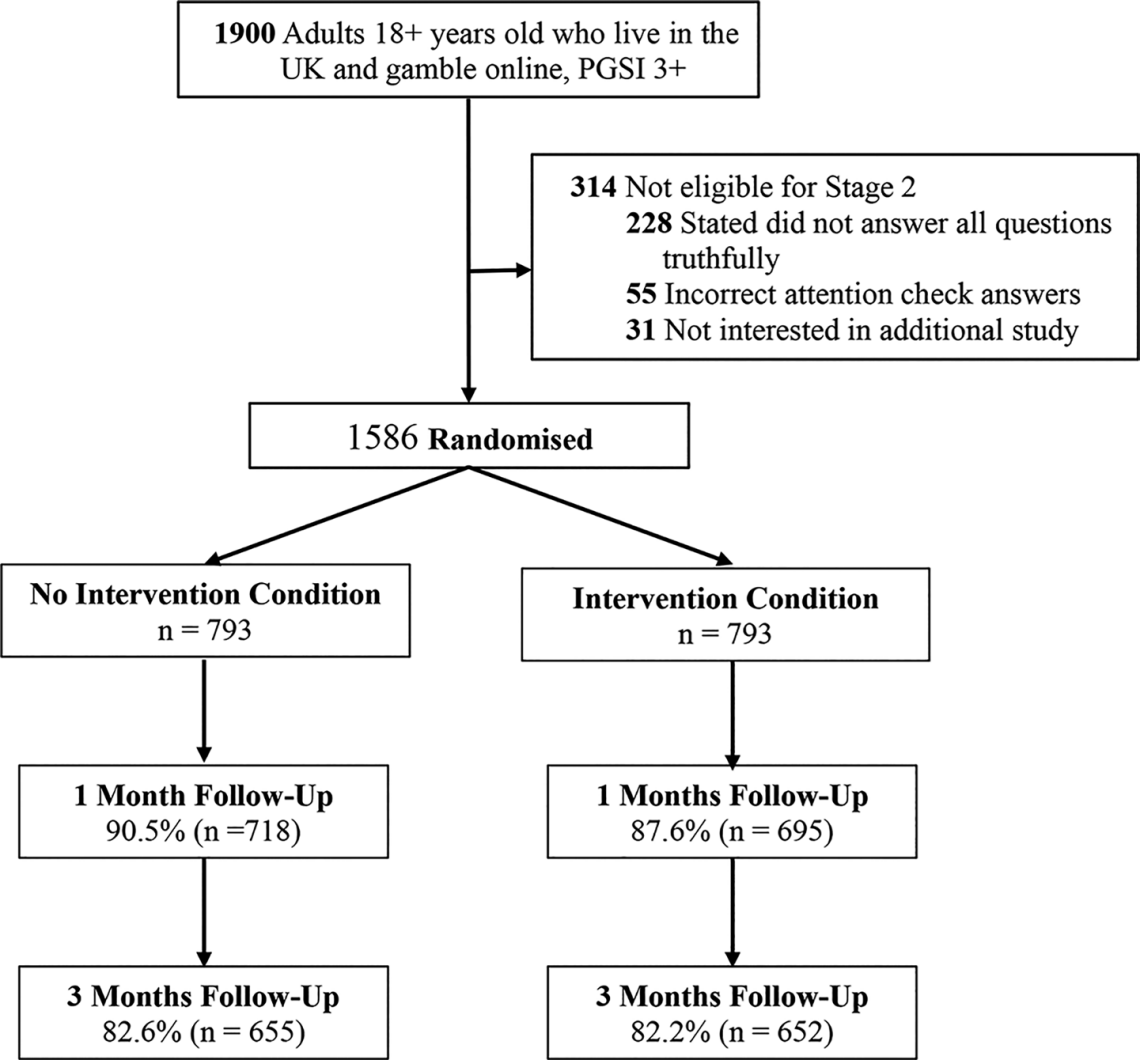
Project flowchart

A total of 1900 surveys with PGSI scores of three or more were completed as part of the first stage of recruitment. Of these, 1586 individuals were eligible for the RCT and participants were equally divided between the Intervention and No Intervention condition (*n* = 793). Follow-up rates were excellent with 89.1% of participants completing the 1-month follow-up survey and 82.4% completing the 3-month follow-up survey. More details, including follow-up information for each condition, can be found in the project flowchart in Fig. 1.

### Sample Characteristics

The average age of the sample was 35.8 years (SD = 10.7), slightly more than half were male (58.9%) and 59.5% had post-secondary education. The majority of participants were full-time, part-time or self-employed (83.4%) and nearly 30% reported a family income under £30,000. With respect to the gambling measures at baseline, the average PGSI was 7.3 (SD = 5.2) indicating at least a moderate level of problems and an increased risk of experiencing consequences related to gambling. The sample reported experiencing an average of 4.3 (SD = 2.9) harms and 4.5 (SD = 3.2) urge related symptoms, as measured by the SGHS and G-SAS, respectively. Only 6.3% reported ever attending formal treatment. See Supplementary Table 1 for more details.

Participants also estimated the average amount of money and time they spent gambling in a typical month and during a typical session, over the past 3 months and how this compared to others’ their same age and gender (normative estimates). In a typical month, participants reported spending an average of 19.4 (SD = 21.7) hours gambling with an average loss of £61.4 (SD = 206.9). Participants estimated 13.1 (SD = 15.0) hours for their peers with an average loss of £62.6 (SD = 86.1). Additional details and session estimates are summarised in Table 1.

**Table 1:** Average estimates of time and money spent by participants in a typical month and session and normative estimates for others of the same age and gender (*n* = 1586)

Outcome Variable		Group	Mean (SD)	Median (range)
Typical Expenditure (GBP)	Month	Participant Report	-61.4 (206.9)	-40 (-451 to 351)
		Normative Estimate	-62.6 (86.1)	-50 (-251 to 121)
	Session	Participant Report	-12.7 (38.2)	-10 (-91 to 61)
		Normative Estimate	-15.2 (18.2)	-10 (-51 to 15)
Typical Time Spent (hours)	Month	Participant Report	19.4 (21.7)	10 (0 to 71)
		Normative Estimate	13.1 (15.0)	6 (0 to 49)
	Session	Participant Report	2.0 (1.8)	0 (0 to 7.1)
		Normative Estimate	1.6 (1.5)	1 (0 to 5)

### Gambling Outcomes

Six separate linear mixed effects models were conducted to investigate the effect of the Intervention on outcome measures compared to the No Intervention condition over time. For all models, there were no significant interactions (*p* > 0.05). That is, none of the gambling outcomes differed over time between the two conditions. With respect to main effects, there were also no significant differences between conditions; however, there were significant main effects of time. In all models, the gambling outcomes showed significant differences between baseline and each follow-up (i.e. 1-month, 3-months). The details of the mixed effects results are displayed in Table 2.

**Table 2:** Mixed-effects models results of time, intervention, and time by intervention on average gambling outcome measures

Predictors	Typical Expenditure (GBP)					
	Month			Session		
	Estimate	t	*p*	Estimate	t	*p*
Intercept	-58.05	-11.03	< 0.001	-13.17	-13.56	< 0.001
Time (Ref: Baseline)						
1-month	42.49	6.69	< 0.001	9.77	7.85	< 0.001
3-months	24.44	3.74	< 0.001	7.73	6.05	< 0.001
Condition (Ref: No Intervention)						
Intervention	-6.49	-0.88	0.381	0.80	0.59	0.556
**Interaction**		***F***	***p***		***F***	***p***
Time by Condition		0.96	0.385		0.99	0.371
**Predictors**	**Typical Time Spent (hours)**					
	**Month**			**Session**		
	**Estimate**	***t***	***p***	**Estimate**	***t***	***p***
Intercept	19.11	31.09	< 0.001	1.97	34.01	< 0.001
Time (Ref: Baseline)						
1-month	-8.76	-14.12	< 0.001	-0.65	-9.46	< 0.001
3-months	-6.51	-10.16	< 0.001	-0.59	-8.30	< 0.001
Condition (Ref: No Intervention)						
Intervention	0.54	0.62	0.535	0.05	0.65	0.514
**Interaction**		***F***	***p***		***F***	***p***
Time by Condition		1.01	0.364		0.47	0.625
**Predictors**	**Short Gambling Harm Screen**			**Gambling Symptom Assessment Scale**		
	**Estimate**	***t***	***p***	**Estimate**	***t***	***p***
Intercept	4.18	40.94	< 0.001	4.42	39.10	< 0.001
Time (Ref: Baseline)						
1-month	-1.40	-14.54	< 0.001	-0.20	-2.01	0.045
3-months	-0.96	-9.69	< 0.001	-0.42	-3.99	< 0.001
Condition (Ref: No Intervention)						
Intervention	0.18	1.26	0.209	0.27	1.67	0.094
**Interaction**		***F***	***p***		***F***	***p***
Time by Condition		1.18	0.307		0.57	0.563

Table 3 reports the estimated marginal means of each gambling outcome for each condition at baseline and each follow-up. The results illustrate that all measures change over time, but not between conditions.

**Table 3:** Estimated marginal means of gambling outcome measures for condition assignment by time

Outcome variable		Condition assignment	Time		
			Baseline mean (SE)	1-Month mean (SE)	3-Months mean (SE)
Typical Expenditure (GBP)	Month	Intervention	-64.55 (5.21)	-16.39 (5.51)	-27.37 (5.66)
		No Intervention	-58.05 (5.26)	-15.56 (5.44)	-33.61 (5.63)
	Session	Intervention	-12.36 (0.96)	-4.84 (1.02)	-4.74 (1.05)
		No Intervention	-13.17 (0.97)	-3.40 (1.01)	-5.44 (1.04)
Typical Time Spent (hours)	Month	Intervention	19.65 (0.62)	9.64 (0.64)	12.63 (0.66)
		No Intervention	19.12 (0.62)	10.35 (0.64)	12.61 (0.66)
	Session	Intervention	2.02 (0.06)	1.28 (0.06)	1.41 (0.06)
		No Intervention	1.97 (0.06)	1.32 (0.06)	1.38 (0.06)
Short Gambling Harm Screen		Intervention	4.37 (0.10)	2.89 (0.11)	3.19 (0.11)
		No Intervention	4.18 (0.10)	2.78 (0.11)	3.22 (0.11)
Gambling Symptom Assessment Scale		Intervention	4.68 (0.11)	4.34 (0.12)	4.15 (0.12)
		No Intervention	4.42 (0.11)	4.21 (0.12)	4.00 (0.12)

Finally, in order to determine if initial gambling severity influenced the results and control for individual differences, all models were repeated including a dichotomous variable that indicated severe gambling at baseline (PGSI ≥ 7) as a fixed effect. While there was some improvement in model fit as indicated by Akaike Information Criterion and Bayesian information values, the significance of the interactions did not change and the effects on other estimates were marginal. Therefore, the simpler models, which do not include the baseline severity fixed effect, are reported.

### Normative Perceptions

Four linear mixed-effects models investigated the effect the Intervention had on changes in participant’s estimates of other people’s gambling compared to the No Intervention group. There were no significant (*p* > 0.05) interactions indicating no difference in estimates over time between the two conditions. There were also no significant main effects of the condition, however, there was a significant change over time in all the estimates, except for time spent in a typical session and between baseline and 1-month estimates of the average amount of money spent in a typical month. Findings are summarised in Table 4.

**Table 4:** Mixed-effects models results of time, intervention, and time by intervention on average normative estimates of gambling outcome measures (previous 3-months)

Predictors	Typical Expenditure (GBP)					
	Month			Session		
	Estimate	t	*p*	Estimate	t	*p*
Intercept	-62.35	-22.01	< 0.001	-15.52	-23.85	< 0.001
Time (Ref: Baseline)						
1-month	4.07	1.22	0.224	2.17	2.71	0.007
3-months	15.76	4.59	< 0.001	2.25	2.75	0.006
Condition (Ref: No Intervention)						
Intervention	0.46	0.12	0.908	0.52	0.57	0.568
**Interaction**		***F***	***p***		***F***	***p***
Time by Condition		1.41	0.246		0.37	0.689
**Predictors**	**Typical Time Spent (hours)**					
	**Month**			**Session**		
	**Estimate**	***t***	***p***	**Estimate**	***t***	***p***
Intercept	13.36	32.06	< 0.001	1.63	32.04	< 0.001
Time (Ref: Baseline)						
1-month	-3.69	-7.83	< 0.001	-0.06	-1.01	0.312
3-months	-3.92	-8.08	< 0.001	-0.08	-1.30	0.193
Condition (Ref: No Intervention)						
Intervention	-0.58	-0.99	0.323	-0.01	-0.13	0.896
**Interaction**		***F***	***p***		***F***	***p***
Time by Condition		0.37	0.691		2.29	0.101

The estimated marginal means calculated based on each model show no differences between conditions, but illustrate the changes found in some of the estimates over time. All means are displayed in Table 5.

**Table 5:** Estimated marginal means of normative estimates for condition assignment by time

Outcome Variable		Condition Assignment		Time		
				Baseline mean (SE)	1-Month mean (SE)	3-Months mean (SE)
Typical Expenditure (GBP)	Month		Intervention	-61.89 (2.83)	-54.22 (3.02)	-37.99 (3.05)
			No Intervention	-62.35 (2.83)	-58.28 (2.95)	-46.59 (3.05)
	Session		Intervention	-15.00 (0.65)	-12.16 (0.70)	-11.78 (0.70)
			No Intervention	-15.52 (0.65)	-13.35 (0.68)	-13.27 (0.70)
Typical Time Spent (hours)	Month		Intervention	12.78 (0.42)	8.90 (0.44)	9.26 (0.45)
			No Intervention	13.36 (0.42)	9.68 (0.43)	9.44 (0.45)
	Session		Intervention	1.62 (0.05)	1.41 (0.05)	1.56 (0.06)
			No Intervention	1.63 (0.05)	1.57 (0.05)	1.55 (0.06)

### Intervention Feedback

Participants ranked each feedback section on a four-point Likert scale (0= “Not at All Useful”; 4= “Very Useful”). Overall, the majority of respondents rated the sections as “Very Useful” (32.0%) and only a small number indicated the sections were “Not at All Useful” (7.1%). Combined, this suggests a strong perception of utility with most participants reporting some benefit from the intervention. A detailed summary of ratings by feedback section can be found in Supplementary Table 2.

Of the 793 participants in the Intervention group, 123 individuals responded to the open-ended question “Do you have any impressions that you would like to let us know about the feedback materials?” (i.e. nothing, no, N/A responses were excluded). Responses were coded as positive (59.3%), neutral (17.1%), negative (8.9%), or suggested changes (14.6%), then reviewed and subcategorised based on emerging consensus and dissent.

The majority of impressions were positive. A high number of responses related to participants reporting an increased awareness of gambling harm and gambling related issues. The information was considered “insightful” and “eye-opening” and participants expressed appreciation for the informative nature. Other participants expressed a shift in perspective and intentions towards gambling, while others recognised the impact of societal norms and media influences on their perceptions and highlighted the survey’s role in fostering self-awareness, prompting reflection, and motivating participants to reassess their relationship with gambling. Some participants reported behavioural changes where they reduced or stopped their gambling. Participants attributed their progress to insights gained from the survey with the majority acknowledging ongoing efforts to reduce or manage their gambling habits. Finally, a few participants considered the material to be useful to others and specifically mention the benefit for individuals who may struggle to self-identify problem gambling behaviours.

The second largest category of responses were neutral with a group of participants struggling to recall specific details or impressions of the material. A number of responses related to individuals providing contextual information and expressed a range of attitudes towards their gambling behaviours, with some acknowledging a difference between their habits and societal norms but not perceiving them as problematic.

A minority of responses were coded as negative and all related to participants finding the material not useful or relevant to them. The feedback reflected skepticism or perceived lack of usefulness of the materials in addressing their gambling habits. Participants cited a lack of utility for individuals with more severe problem gambling and those with very low levels of gambling.

Some participants gave feedback on specific survey elements they either found especially supportive or would like to see improved. Suggestions included changing the measures used in the survey (considering past gambling as well as current gambling), feedback (more priority given to monetary impact of gambling) and inclusion of motivations or “why” individuals gamble. Further to this, two individuals expressed concern over the accuracy and validity of the feedback. A small number of participants identified preferences for a more straightforward and less patronizing tone in the feedback materials. Broadly, participants expressed preference for visual representations of data and information in the feedback materials. While the infographic style was appreciated, some noted struggling to take-in the numerical information and the benefit of future graphical displays was noted.

## Discussion

This project sought to determine the usability of the modified, online gambling intervention and evaluate if it helped decrease short-term gambling and altered normative perceptions. The RCT did not detect a significant difference between participants who received or did not receive the intervention, however, participants reported significant reductions in both the money and time they spent gambling in a typical session and in a typical month, experienced fewer harms, and reported fewer urges to gamble at both follow-ups compared to baseline, indicating a main effect of time.

Individuals often overestimate how frequently others their age and gender participate in similar activities. Correcting these misperceptions by providing actual population estimates can lead individuals to change their behaviours to align more closely with others. In the current sample, nearly all of the participants (99%) estimated that others their same age and gender gambled in the last year, while findings from the 2016 National Health Survey reported 43% do not gamble at all (Conolly et al., 2018).

Normative feedback interventions have been found to impact a number of different behaviours, however effect sizes are generally small (Bennett et al., 2020). However, given the large sample size employed in this study, there was power to detect a small difference between groups. It is possible that the intervention, in its current form, is not effective. While the intervention was adapted from a previously tested version and received generally positive feedback from participants, it did not appear to have produced behavioural effects beyond those achieved through the act of self-monitoring or participation in the study process alone.

Another potential explanation for the lack of group differences is that participants in the control group were asked to reflect on intervention components, which may have prompted self-regulatory thought processes similar to those elicited in the intervention group. This non-intervention condition, though not designed to be therapeutic, may have had an unintended effect. Similarly, participants in both groups may have responded to the act of completing repeated gambling assessments and engaging with content that prompted reflection. Furthermore, because the intervention was delivered universally to individuals scoring 3 or above on the PGSI regardless of their motivation to change, it is possible that effects were diluted by a lack of readiness to reduce gambling among some participants. While similar interventions have demonstrated modest effects in treatment-seeking and student populations, it may be necessary to tailor the intervention more specifically to readiness to change or to add motivational enhancement elements in future iterations. Another related issue to consider is that participants recruited through online panels may be participating in the trial more out of a desire to earn funds than because of a concern about their gambling (Cunningham et al., 2019). Taken together, these findings highlight that although internet-based normative feedback interventions are promising in terms of reach and acceptability, further refinement, at least for the current intervention, is necessary to strengthen any potential impact on gambling behaviour. These refinements may include more targeted recruitment, personalised tailoring of feedback, or integration of motivational and interactive components.

The ability of the Internet to reach large numbers of people contributes to the significant potential of these interventions as a small change multiplied across a large group could still result in notable reductions in gambling behaviour, related harms, and consequences. In turn, this could improve health related outcomes for individuals, families, communities, and the general population. There is also growing evidence that delivering brief-interventions online can support behaviour change (Bennett et al., 2020) making them a feasible alternative to traditional treatment. Such interventions are cost-effective to deliver and the widespread availability and use of the Internet makes them easily scalable. This creates an opportunity to connect a large proportion of the population with efficacious means to address health concerns. They also create an opportunity to support individuals who may not otherwise seek formal treatment. Indeed, nearly 94% of this sample reported never seeking help for their gambling. This is particularly concerning as, at baseline, over a third (36.8%) of these participants also had PGSI scores indicating moderate or problem gambling and an increased risk of experiencing negative consequences.

Overall, the feedback received from participants about the intervention was positive. Online interventions are generally well-received by users which is another advantage of offering treatment options via the Internet (Sagoe et al., 2021). However, a minority of participants found the feedback unhelpful or not relevant to their situation. In particular, these participants felt that the feedback would not be useful for those with very low or very high levels of gambling. Some participants expressed concern about the tone of the feedback, describing it as overly directive or insufficiently engaging. Notably, several participants recommended adding normative feedback about how average spending compares to others. Unfortunately, no national surveys currently collect information on gambling expenditures (Rochester & Cunningham, 2022). In addition to allowing the addition of normative feedback regarding financial expenditures in interventions such as the present one, collecting such information would provide another means to monitor the risk of harms in the population and it is recommended that questions collecting information related to spending be added to future national gambling surveys.

In conclusion, the large number of at-risk individuals in this sample who have never sought treatment demonstrates the need for alternatives to traditional treatment and the misperceptions observed in this sample support the use of normative feedback interventions as an avenue to try and effect this change. The Internet is a promising medium for providing large scale, publicly available, self-directed interventions to help reduce gambling prevalence, risk, and related harms.

## Electronic Supplementary Material

Below is the link to the electronic supplementary material.

Supplementary Material 1

## Data Availability

Data is available from the corresponding author on reasonable request.
