# Supplementary material for: Pilot Randomised Trial of a Brief Online Personalised Feedback Intervention for the UK Context Designed To Prevent, Reduce, and Address Gambling Harm
Source: J Gambl Stud. 2025 Jun 27;41(3):977–91. doi: 10.1007/s10899-025-10401-2 (PMC12360968; doi:10.1007/s10899-025-10401-2)
Supplement: Supplementary file 1 — Supplementary Material 1 [file 10899_2025_10401_MOESM1_ESM.docx]

**Supplementary Table 1.** Demographics and baseline clinical characteristics of full sample (n=1586)

| **Demographics** | **% (n)** |
| --- | --- |
| Age, mean years (SD) | 35.8 (10.7) |
| Males | 58.9 (934) |
| Post-Secondary Education | 59.5 (943) |
| Married/Common law/Same sex partner | 50.2 (796) |
| Full/Part-time/Self-employed | 83.4 (1323) |
| Family Income < £30,000 (missing n=29) | 29.9 (474) |
| Ever attended formal treatment (missing n=5) | 6.3 (99) |
| **Clinical Characteristics** | **mean (SD)** |
| Problem Gambling Severity Index (PGSI) | 7.3 (5.2) |
| Short Gambling Harm Screen (SGHS) | 4.3 (2.9) |
| Gambling Symptom Assessment Scale (G-SAS) | 4.5 (3.2) |

**Supplementary Table 2.** Utility Rating by Feedback Section (n=693)

| **Feedback Section** | **Usefulness Rating, % (n)** | | | | |
| --- | --- | --- | --- | --- | --- |
|  | **Very** | **Moderate** | **Slightly** | **Not at All** | **Don’t Know/ Recall** |
| How total number of types of gambling engaged in compares to that of other adults of the same gender | 28.4  (197) | 30.7  (213) | 22.1  (153) | 6.3  (44) | 12.4  (86) |
| Graph showing likelihood of experiencing problems based on the number of different types of gambling | 32.2  (223) | 29.0  (201) | 18.5  (128) | 7.2  (50) | 13.1  (91) |
| Graph showing likelihood of experiencing problems based on gambling frequently | 32.6  (226) | 29.9  (207) | 16.9  (117) | 6.3  (44) | 14.3  (99) |
| Report of the Problem Gambling Index score | 28.1  (195) | 25.1  (174) | 19.6  (136) | 9.5  (66) | 17.6  (122) |
| Suggestions on ways to reduce the risk of harm | 38.4  (266) | 24.4  (169) | 17.3  (120) | 6.2  (43) | 13.7  (95) |
| **Overall** | 32.0  (1107) | 27.8  (964) | 18.9  (654) | 7.1  (247) | 14.2  (493) |
